# Brain functional changes in patients with Crohn's disease: A resting‐state fMRI study

**DOI:** 10.1002/brb3.2243

**Published:** 2021-06-14

**Authors:** Lu Li, Jie Ma, Jian‐Guang Xu, Yan‐Ling Zheng, Qian Xie, Lan Rong, Zong‐Hui Liang

**Affiliations:** ^1^ Department of Radiology, Jing'an District Centre Hospital of Shanghai Fudan University Shanghai China; ^2^ School of Rehabilitation Science Shanghai University of Traditional Chinese Medicine Shanghai China; ^3^ Department of Gastroenterology, Huashan Hospital Fudan University Shanghai China

**Keywords:** amplitude of low‐frequency fluctuations, Crohn's disease, functional connectivity, functional MRI, regional homogeneity

## Abstract

**Background:**

Crohn's disease (CD) is a chronic recurrent intestinal inflammatory disease, often accompanied by poor adaptation and excessive stress response. However, the potential neurological mechanisms of these symptoms have not yet been studied in‐depth.

**Objective:**

To investigate alterations in brain activity in patients with Crohn's disease and study the relationship between altered regions and clinical indices.

**Methods:**

A total of 15 CD patients and 26 matched healthy controls were recruited. All participants underwent fMRI scans. The amplitude of low‐frequency fluctuations (ALFF) and regional homogeneity (ReHo) assessed differences in spontaneous regional brain activity. Differences between the groups were selected as seeds for functional connectivity (FC) analyses. Correlations between disease duration and ALFF/ReHo/FC values in abnormal regions were analyzed.

**Results:**

Patients with CD had significantly higher ALFF values in the left superior frontal gyrus, anterior cingulate cortex, and supplementary motor area, and lower values in the left hippocampus. They also had higher ReHo values in the left anterior cingulate cortex, supplementary motor area, putamen, and the bilateral superior frontal gyri. FC strength in the left precentral and middle temporal gyri was found to be increased when the left superior frontal gyrus was used as the seed point. FC strength was also observed to be increased in the left postcentral, middle frontal gyri, inferior frontal orbital cortex, and right rolandic operculum when the left anterior cingulate cortex was used as the seed point.

**Conclusion:**

CD demonstrated abnormal neural activity and FC in various regions primarily associated with emotional, pain and cognitive‐related functions, which provides more information to further understand the neural mechanisms of the disease.

## Graphical abstract

This study investigated the altered brain activity and connectivity in CD patients by using rs‐fMRI with ALFF, ReHo, FC analysis. CD demonstrated abnormal neural activity and functional connectivity in various regions mainly related to emotion and mood function, which provide more information to further understand of neural mechanisms of the disease.

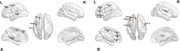

## 1 INTRODUCTION

Crohn's disease (CD) is a chronic recurrent intestinal inflammatory disease. Although its etiology involves factors such as heredity, immunity, environment, and infection, its pathogenesis remains unclear (Baumgart & Carding, 2007). Recent epidemiological research indicates that CD incidence might be rising rapidly in Asia (Ng et al., 2018). The whole gastrointestinal tract can be involved in the disease, typically requiring lifelong medication and supportive care due to relapsing intestinal tract inflammation and recurrent intestinal or extraintestinal symptoms, resulting in poor quality of life (Torres et al., 2017). It is believed that white matter lesions and neurological deficits in patients with inflammatory bowel disease (IBD) may be an extraintestinal manifestation of the disease (Geissler et al., 1995; Morís, 2014). There is also information indicating that the condition may induce psychological stress, precipitating mental disturbances and deficiency in concentration (van Langenberg et al., 2017). The brain‐gut axis is the complex bidirectional communication, which not only maintains gastrointestinal homeostasis but also affects higher cognitive functions, emotion, and motivation. By changing the state of the hypothalamic‐pituitary‐adrenal axis (HPA), the autonomic nervous system (ANS) directly affects the modulation of gut functions. In turn, the input information of the gut and mental stress can activate related brain regions, which mainly include hypothalamus, limbic system and cerebral cortex area (B. L. Bonaz & Bernstein, 2013; Gracie et al., 2018; Gracie et al., 2019). Meanwhile, enteric microbiota is also likely to interact with nervous system via neural signaling, endocrine and immune mechanisms (Carabotti et al., 2015). Studies suggested that vagal nerve stimulation or good psychological status maintenance can reduce the recurrence rate of the disease and even improve the efficiency of remission induction in active stage (B. Bonaz et al., 2016; May, 2008; Mayer et al., 2014).

Numerous studies have reported significant changes in brain structure and activity in CD patients. Agostini, Benuzzi, et al. (2013) reported that CD patients demonstrated reduced gray matter (GM) volume within the frontal lobe and cingulate cortex. Nair et al. (2016) found that patients with CD had significant cortical thickening in the left superior frontal area. Both significantly reduced GM and cortical thicknesses were found by C. H. Bao et al. (2015) involving multiple brain regions related to affective symptoms. However, Kornelsen et al. (2020) found no structural difference between CD patients and controls using voxel‐based morphometry analysis, and their study showed increased FC between the frontal and parietal network and the salience network. In a task‐related study, patients with CD showed decreased activation in the medial temporal lobe, insula, putamen, and cerebellum (Agostini, Filippini, et al., 2013), while patients had increased activation in the midcingulate cortex in another study using the stress‐evoking task (Agostini et al., 2017). Higher reactivity in the inferior occipital gyrus, middle temporal gyrus, superior parietal lobule, and inferior frontal gyrus were also reported in CD patients (Nair et al., 2019). Y. Fan et al. (2020) reported that the intrinsic functional connectivity (FC) between the amygdala and insula, parahippocampus, anterior middle cingulate cortex was decreased, and abnormal FC is associated with the duration of disease. In addition, the intra‐network FC analysis showed significantly increased connectivity within the executive control network and default mode network (DMN; Hou et al., 2019).

To our knowledge, few studies had jointly performed ALFF and ReHo analysis to observe brain activity in CD patients. In the current study, we used resting‐state functional MRI to investigate alterations in brain activity and changes in FC across the CD patients. The amplitude of low‐frequency fluctuations (ALFF) reflects the level of spontaneous activity at each voxel (Y. F. Zang et al., 2007), and regional homogeneity (ReHo) evaluates the degree of synchronization based on fluctuations of BOLD signals among neighboring voxels to provide information about the local activity (Y. Zang et al., 2004). We focused on exploring the aberrant brain activity by combining voxel‐wise ALFF and ReHo calculations, and obtaining information about the functional integrity of brain networks by seed‐based FC in CD patients. We hypothesized that there would be aberrant brain function in emotion or pain‐related processing regions in patients compared with healthy controls (HC), and the neuroimaging changes would be associated with duration of disease, which may provide a better understanding of the underlying neural mechanisms.

## 2 MATERIALS AND METHODS

### 2.1 Subjects

This study was approved by the Ethics Committee of the Shanghai Jing'an Centre Hospital, all subjects signed written informed consent.

Fifteen CD patients and twenty‐six matched HC were enrolled in this study from February 2018 to June 2019. All patients were evaluated by an experienced gastroenterologist. Eligible CD patients who met the inclusion criteria were continuously recruited. Endoscopic and hematological examination data were obtained.

Inclusion criteria: (1) right‐handed; (2) age, 18−55 years; (3) education level, 9 years at the minimum; (4) clinical remission for more than 6 months, assessed upon clinical and biological evaluation (the CD Activity Index [CDAI < 150], and C reactive protein level [CRP < 5 mg/mL]) (Best et al., 1976; Khanna et al., 2015).

Exclusion criteria: (1) intestinal‐related abdominal surgery; (2) use of glucocorticoids, biologics or psychotropic in the previous 6 months; (3) with current or previous history of neurological or psychiatric disease and pain syndromes, psychiatric examinations were assessed by a psychiatrist based on a psychiatric interview tool from the Diagnostic and Statistical Manual of Mental Disorders, Fifth Edition (DSM‐5) (Kocsis, 2013); (4) organic brain lesions and obvious white matter degeneration; (5) MRI scan‐related contraindications such as claustrophobia or metallic implants.

Matched with age, sex, handedness, and education level, the healthy subjects (HCs) were recruited with advertisement. They did not suffer from any digestive or pain‐related diseases, and were negative in colonoscopy examination.

Moreover, patients were phenotyped according to the Montreal classification (Satsangi et al., 2006) to determine location [L1 (ileum), L2 (colonic), L3 (ileocolonic), and L4 (only upper disease)], and disease behavior [B1 (only inflammatory disease), B2 (fibrostenosing disease), B3 (fistula), P (perianal disease)]; disease duration in years was recorded.

### 2.2 MRI data acquisition

MRI data of all participants were acquired on a GE 3.0T magnetic resonance scanner equipped with a standard eight‐channel phased‐array head coil. Participants were asked to avoid caffeine or other similar substances on the day of examination. With the supine position on the scanner, they were instructed to relax, keep their eyes closed, and stay awake while avoiding systematic cognitive or motor activity during scanning. A sponge pad and earplugs were used to prevent head movement and protect the ears from the scanner noise. Functional images were acquired with a single‐shot gradient‐recalled echo planar imaging (EPI) sequence using the following parameters: time points: 240; slices: 41; slice thickness: 3 mm, slice gap: 3 mm; TR: 2000 ms; TE: 30 ms; FA: 90^◦^; FOV: 256 × 256 mm; and matrix size: 64 × 64; in‐plane resolution: 3.75 mm × 3.75 mm.

### 2.3 Imaging data preprocessing

The imaging data processing package RESTplus1.2 (Jia et al., 2019) was used to analyze the rs‐fMRI data, which is based on Statistical Parametric Mapping 12 (SPM 12, http://www.fil.ion.ucl.ac.uk/spm) and MATLAB (2014a). The preprocessing steps are as follows: (1) slice scan time correction, (2) head movement correction (the head movements were less than 2.5 mm or 2.5° in any direction), (3) spatial normalization of the functional images via the standard EPI template (Calhoun et al., 2017), (4) spatial smoothing using a Gaussian kernel with a full‐width at half‐maximum (FWHM) of 6 mm × 6 mm × 6 mm (to avoid spurious connections, smoothing will apply after ReHo analysis), and (5) removal of linear trends.

### 2.4 ALFF analysis

The time series were transformed into the frequency domain using a fast Fourier transform, and the square root of the power spectrum was calculated and averaged across 0.01–0.08 Hz within each voxel. Subsequently, the ALFF of each voxel was divided by the global mean ALFF value to standardize data across subjects.

### 2.5 ReHo analysis

After preprocessing, ReHo maps were produced for each participant based on Kendall's coefficient of concordance (KCC) of the given voxel time series with its nearest 26 neighbors. Finally, the generated images were spatially smoothed with a 6‐mm FWHM Gaussian kernel.

### 2.6 Seed‐based FC analysis

Based on the ALFF and ReHo findings, the changed brain regions (voxel size > 40, and key regions in previous studies were taken into consideration) in CD compared with normal control groups, were chosen as seeds. By using RESTplus software, a 6 mm radius around the peak point was taken to encompass the regions of interest (ROIs) for the seed‐based FC analysis. Pearson correlation coefficients were calculated between the time courses of seed regions and the time series of all voxels in the global brain. Finally, Fisher's r‐to‐z transformation was applied to all maps before statistical analysis.

### 2.7 Statistical analysis

Demographic and clinical measurements were compared between groups using IBM SPSS Statistics for Windows, Version 20.0 (IBM Corp., Armonk, NY, USA), and the independent sample *t*‐test was used for continuous variables, and chi‐square test for the sex proportion, which was the categorical variable. In addition, variables that did not conform to normal distribution were analyzed using the Mann‐Whitney rank test, and *p* < 0.05 was considered statistically significant.

For ALFF, ReHo, and FC analyses, the two‐sample t‐test of the RESTplus1.2 statistical module was utilized. Family wise error (FWE) was used to correct multiple comparisons using SPM12. A statistical significance of *p* < 0.05 at cluster level was set. During the calculations, age, gender, and education level were used as covariates.

In the CD group, the mean ALFF, ReHo values, and mean FC z‐values were extracted from the regions with significant group differences. Pearson's linear correlation analyses were then applied to assess the relationships between CD disease duration and ALFF, ReHo, and FC strength. Calculations were performed using SPSS after controlling the influences of age and sex. Statistical significance was set at *p* < 0.05(Bonferroni correction).

## 3 RESULTS

### 3.1 Clinical and demographic characteristics

Demographic and clinical characteristics of the CD and HC groups and the comparison between them are shown in Table 1. The two groups did not differ significantly in terms of age, gender, and education level.

**TABLE 1:** Demographics, clinical data between CD and HC group

Characteristics	CD patients (*n* = 15)	HCs (*n* = 26)	*p*‐Value
Age (years)	34.20 ± 13.07	30.38 ± 9.98	0.209^a^
Gender (male/female)	11/4	19/7	0.890^b^
Education (years)	15.40 ± 2.72	15.19 ± 2.23	0.287^a^
Disease duration (years)	4.57 ± 3.90		
Montreal classification	L1:L2:L3:L4 = 9:2:3:1		
	B1:B2:B3:P = 9:4:1:1		

*Note*: L1, ileum; L2, colonic; L3, ileocolonic; L4, upper gastrointestinal tract disease; B1, only inflammatory; B2, fibrostenosing in bowel; B3, fistula; P, perianal disease.

^a^ Two sample *t*‐test.

^b^Chi square test.

Abbreviations: CD, Crohn's disease; HCs, healthy controls.

### 3.2 ALFF analysis

**FIGURE 1:**
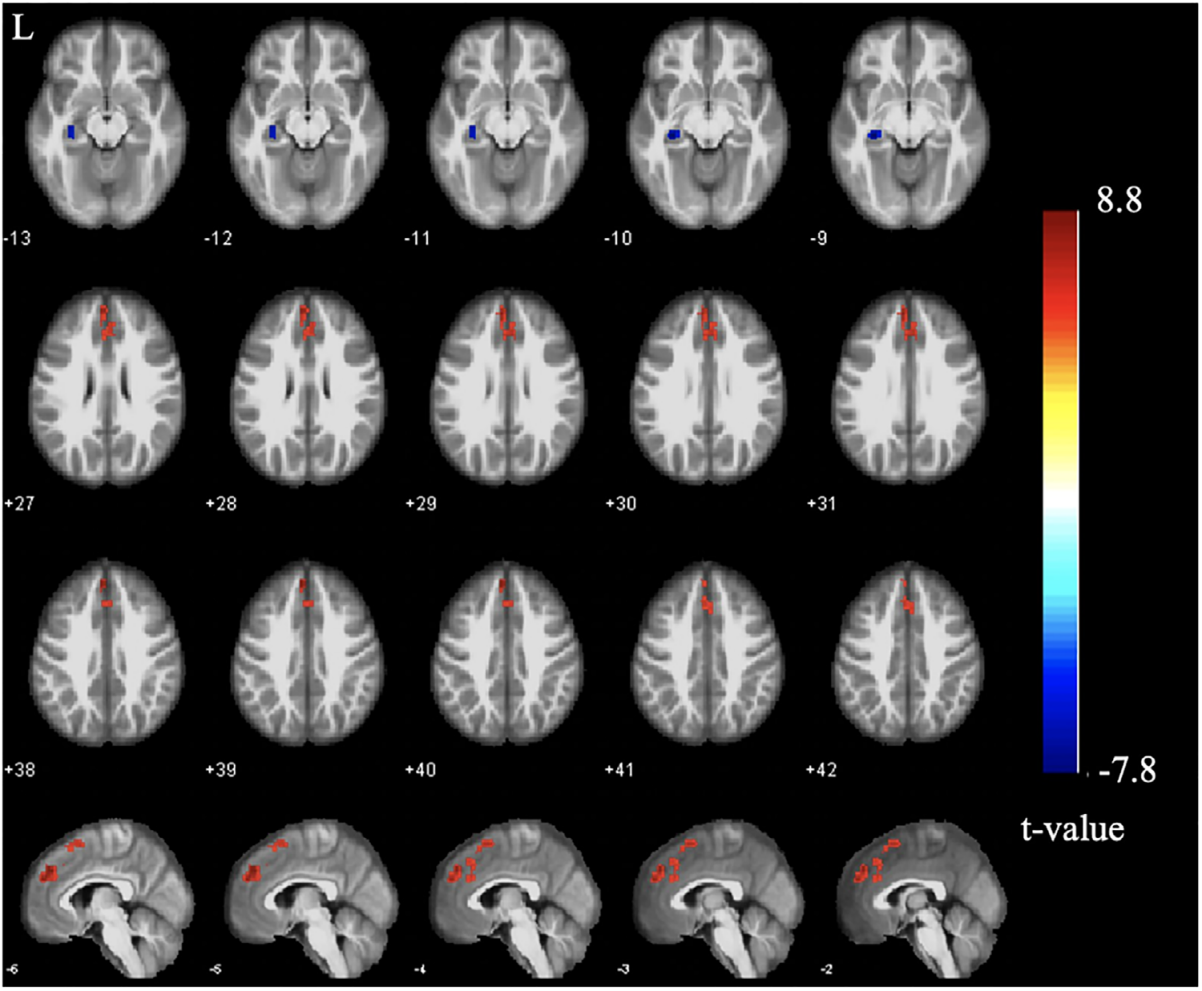
ALFF differences between groups. Significantly increased (red) and decreased (blue) ALFF values in patients with remissive CD compared with healthy controls (FWE‐corrected *p* < 0.05, cluster level). The color bar represents the *t*‐value of the two‐sample *t*‐test between the two groups. Abbreviations: ALFF, amplitude of low‐frequency fluctuation; CD, Crohn's disease; L, left.

The CD group showed significantly higher ALFF values in the left anterior cingulate cortex, left superior frontal gyrus, and left supplementary motor cortex, and lower ALFF in the left hippocampus, when compared with the HC group (FWE‐corrected, cluster level *p* < 0.05; Table 2; Figure 1).

**TABLE 2:** ALFF alterations between the CD and HC group

	Main brain region	Voxel size	*t*‐value	MNI peak point coordinates			
				*x*	*y*	*z*	Seed
Positive	Medial superior frontal gyrus_L	126	8.743	−6	42	36	1
	Anterior cingulate_L	48	6.42	−3	33	27	2
	Supplementary motor area_L	126	6.528	3	24	45	
Negative	Hippocampus_L	32	−7.775	−30	−27	−9	

*Note*: Positive, CD group>HCs group; Negative, CD group<HCs group; CD, Crohn's disease; HCs, healthy controls.

Abbreviations: L, left; R, right.

### 3.3 ReHo analysis

**FIGURE 2:**
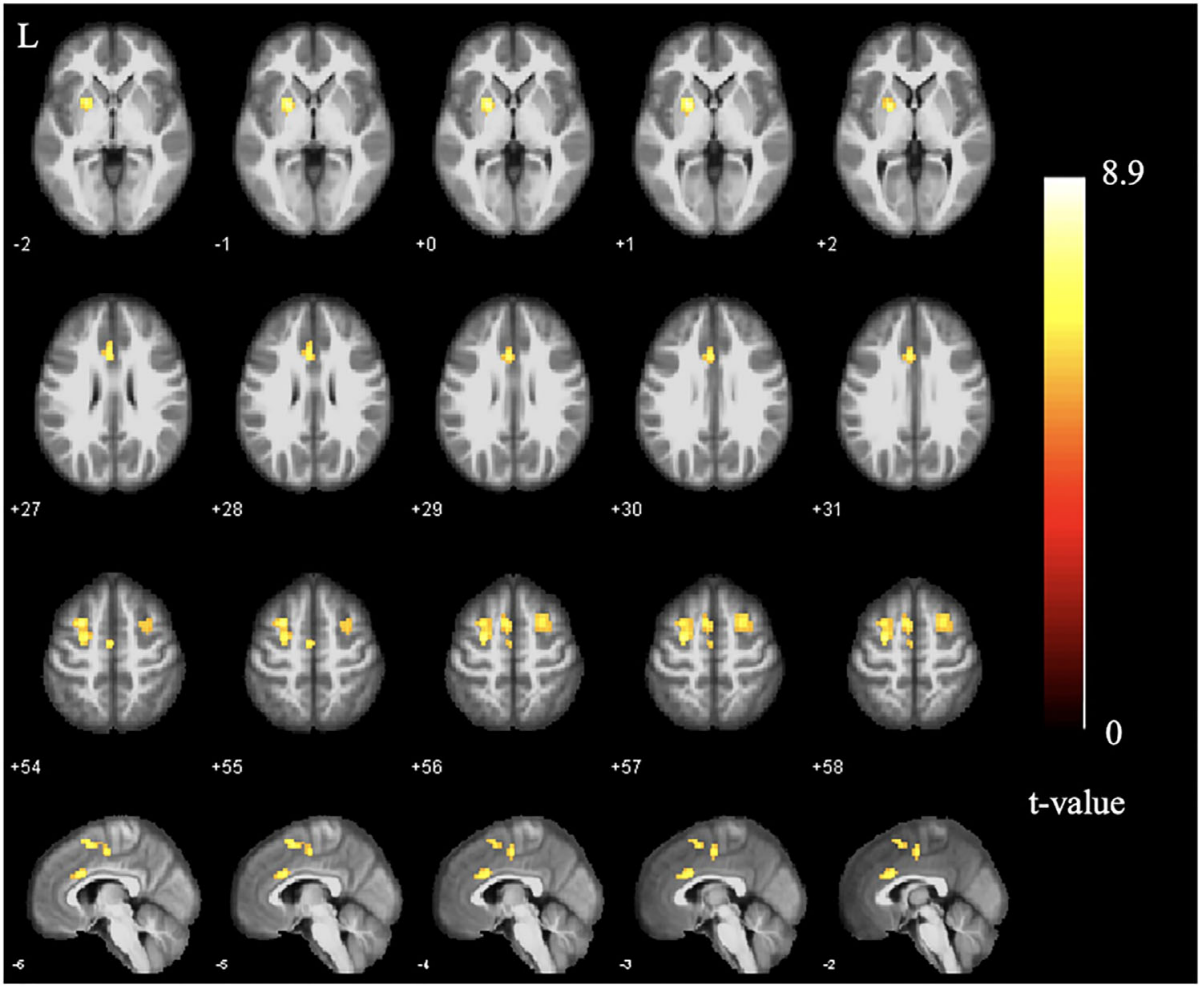
ReHo differences between groups. Significantly increased (yellow) ReHo values in patients with CD compared with healthy controls (FWE‐corrected *p* < 0.05, cluster level). The color bar represents the *t*‐value of the two‐sample *t*‐test between the two groups. Abbreviations: CD, Crohn's disease; L, left; ReHo, regional homogeneity.

The CD group showed significantly higher ReHo values in the left anterior cingulate cortex, bilateral superior frontal gyrus, left supplementary motor cortex, and left putamen, when compared with the HC group (FWE‐corrected, cluster level *p* < 0.05; Table 3; Figure 2).

**TABLE 3:** ReHo alterations between the CD and HC group

	Main brain region	Voxel size	t‐value	MNI peak point coordinates		
				*x*	*y*	*z*
Positive)CD group>HCs group(	Superior frontal gyrus_L	81	7.70	−27	−3	57
	Superior frontal gyrus_R	62	7.35	24	6	60
	Putamen_L	47	8.89	−24	6	0
	Anterior cingulate_L	43	7.46	−6	18	30
	Supplementary motor area_L	76	7.33	−6	9	60

Abbreviations: CD, Crohn's disease; HCs, healthy controls; L, left; R, right.

### 3.4 FC analysis

**FIGURE 3:**
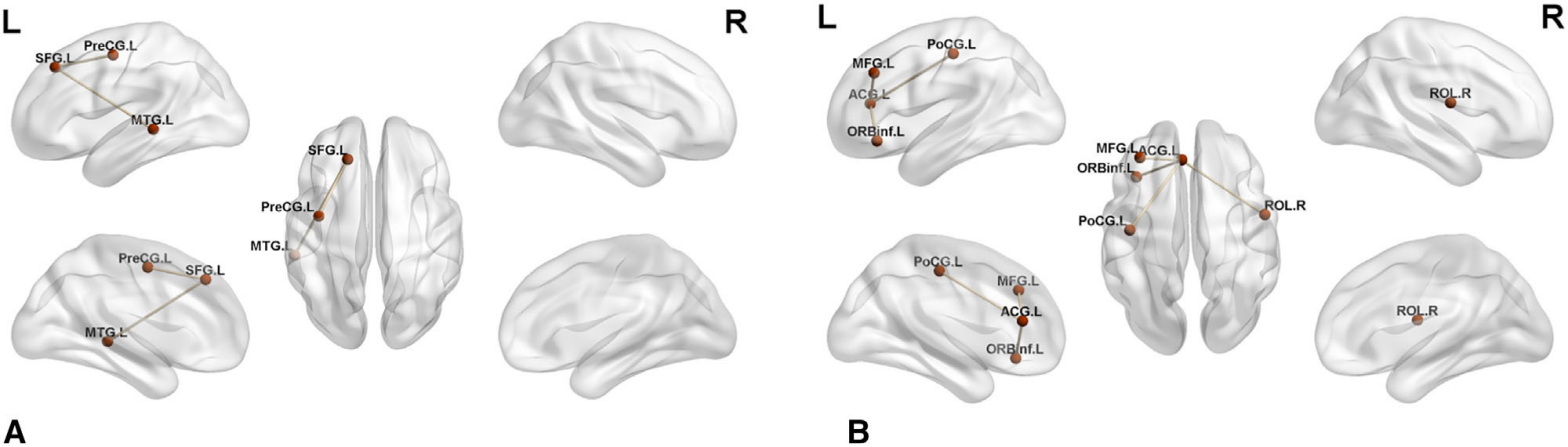
(a) Results of FC analysis selecting SFG.L as seed point. Significant brain regions showing increased functional connections of the SFG.L between CD patients and HCs (*p* < 0.05, FEW‐correction); (b) Results of FC analysis selecting ACG.L as seed point. Significant brain regions showing increased functional connections of the ACG.L between CD patients and HCs (*p* < 0.05, FEW‐correction) Abbreviations: ACG.L, left anterior cingulum; L, left; MFG.L, left middle frontal gyrus; MTG.L, left middle temporal gyrus; ORBinf.L, left inferior frontal orbital cortex; PoCG.L, left postcentral gyrus; PreCG.L, left precentral gyrus; R, right; ROL.R, right rolandic operculum; SFG.L, left superior frontal gyrus.

Based on the ALFF and ReHo results, two peak points, including the left superior frontal gyrus (SFG.L) and left anterior cingulate cortex (ACG.L), were chosen as seed points. Using the left superior frontal gyrus as the seed, the strengths of the FC in the left precentral gyrus and left middle temporal gyrus was found to be increased in CD patients compared with the controls. By selecting the anterior cingulate cortex as the seed, the strengths of the FC in the left postcentral gyrus, inferior frontal orbital cortex, middle frontal gyri, and right rolandic operculum, was observed to be increased in the CD group (Table 4, Figure 3a,b).

**TABLE 4:** FC alteration between the CD and HC group

Connected region	Peak areas	Voxel size	t‐value	MNI peak point coordinates		
				*x*	*y*	*z*
Seed 1 SFG.L	Precentral gyrus.L	50	7.89	−18	−21	69
Positive	Middle temporal gyrus.L	45	6.86	−45	−63	9
Seed 2 ACG.L	Postcentral gyrus.L	72	7.66	−36	−27	45
Positive	Inferior frontal orbital cortex.L	77	7.06	−54	−18	42
	Rolandic operculum.R	40	6.92	63	−12	12
	Middle frontal gyrus.L	38	6.64	−36	42	21

*Note*: Positive, CD group>HCs group; CD, Crohn's disease; HCs, healthy controls.

Abbreviations: ACG, anterior Cingulum; L, left; R, right; SFG, superior frontal gyrus.

### 3.5 Correlation among clinical data, ALFF, ReHo and FC values

There were no significant correlations between the ALFF/ ReHo and FC of aberrant regions and time duration (*p* > 0.05, Bonferroni correction).

## 4 DISCUSSION

In this resting‐state fMRI study, we combined ALFF, ReHo, and FC methods to identify alterations in neuronal activity and FC across the whole brain in CD patients. The current study found that CD patients had higher ALFF values in the left anterior cingulate, left medial superior frontal gyrus, left supplementary motor area, and lower ALFF values in the left hippocampus. ReHo values also significantly increased in the left anterior cingulate cortex, bilateral superior frontal gyrus, left supplementary motor area, and left putamen.

First of all, in this study, the brain regions with spontaneous activity differences include some areas related to emotion, pain, and sensorimotor. The specific brain regions are superior frontal gyrus, anterior cingulate gyrus, hippocampus, putamen, and supplementary motor area. The frontal lobe is related to emotional regulation, receives input from the limbic system, and plays an essential role in emotional memory storage (Bermpohl et al., 2006). Studies have shown that the development of children's frontal lobe function is vital for their emotional intelligence (Rosso et al., 2004). Remitted depression patients showed abnormal activation in the prefrontal region when exposed to a negative emotional stimulus compared to the activation observed in a normal control group (Kerestes et al., 2012), and there is evidence that changes in the frontal cortex are involved in its role in anti‐injury and pain regulation (Ong et al., 2019). The functional changes of the superior frontal gyrus have also been reported in IBD patients (Anne Kerstin Thomann et al., 2020). In line with previous findings, our results indicated that patients have increased activity in emotional processing regions, explaining some patients'overreactions when dealing with negative emotions.

The cingulate cortex was considered directly related to the internal neural network of the gastrointestinal tract (Agostini et al., 2011; Vogt, 2013). A recent meta‐analysis on structural and functional brain changes in CD exhibited reduced brain connectivity in cingulate cortex and paracentral lobule (Yeung, 2020). fMRI analysis found that multiple brain regions were activated under a high‐pressure task in CD patients, including the middle cingulate cortex, which revealed differences in stress adaptation (Agostini et al., 2017). The anterior cingulate gyrus has multiple functions, including emotions, cognition, movement, visceral movement, and social interaction (Davis et al., 2005; Price, 2000), and abnormal activation in the frontal lobe and anterior cingulate cortex were also found in irritable bowel syndrome patients (Song et al., 2006). It is believed that the cingulate gyrus can initiate and regulate autonomic neuroendocrine reactions and control inflammatory activities (Gracie et al., 2018). The supplementary motor area is a part of a cortical network involved in the translation of painful cognition (Nachev et al., 2008), and putamen participates in advanced cognitive functions such as memory, emotion, and reward learning. These changed regions may be associated with symptoms of CD sensorimotor (Kimura et al., 1993; Packard & Knowlton, 2002). Increased activity in these areas may be a manifestation of high sensitivity of sensation and the basis for rapid response to stimulus.

Through the regulation of the hypothalamus, the hippocampus plays a vital role in stress suppression, chronic intestinal inflammation affects hippocampal neurogenesis, and its reduction may be the basis of the behavior of IBD patients (Zonis et al., 2015). There are also differences in neurochemistry and perfusion in patients with CD. MRS analysis showed that the ratio of glutamine concentration to total creatine in patients with CD decreased, and the regional cerebral blood flow in CD patients increased significantly. Glutamate is the primary excitatory neurotransmitter in the brain, whose receptor is mainly expressed in the hippocampus, participating in many brain functions, including memory and emotion process (Hao et al., 2016; Riedel et al., 2003). In our study, the activity of the hippocampus was reduced. Similar results were also demonstrated in UC patients (W. Fan et al., 2019). However, in a study by C. Bao et al. (2018), CD patients showed higher ALFF values in the hippocampus and parahippocampus. Although inflammation is relatively quiescent in patients with CD in remission, the relapsing‐remitting disease course and disease behavior might impact functional activities of the brain, leading to inconsistent activity in the same region between researches. We speculated that the hippocampus is affected in earlier stage, or the hippocampus is more sensitive to noxious stimulus, leading to irreversible destruction to the internal function as the disease progresses.

Additionally, FC analysis was performed based on altered ALFF and ReHo values in the left superior frontal gyrus and left anterior cingulate, which may further indicate the potential adaptive function of connective networks in CD patients. In the patient group, the left superior frontal gyrus showed higher FC with the left precentral and left middle temporal gyri, and left anterior cingulate gyrus showed higher FC with the left postcentral gyrus, left inferior frontal orbital cortex, left middle frontal gyrus, and right rolandic operculum.

The superior frontal gyrus and anterior cingulate gyrus, which engages in the stress and emotional regulation of pain (Vogt, 2005), are components of the DMN. A recent study also suggested that the FC was increased between the right precuneus and right posterior cingulate cortex in DMN among CD patients (Hou et al., 2019; Kornelsen et al., 2020). Thomann et al. (2017) exhibited increased connectivity in the anterior cingulate and left superior medial frontal gyrus (aDMN). Precentral and postcentral gyrus were the vital part of sensorimotor networks, which was considered areas of pain‐related sensation. The sensorimotor network also reported higher FC in chronic low back pain (Shen et al., 2019). Temporal lobe is related to language processing (Wagner et al., 2014). Dancey et al. (2009) showed that compared to HC and controls with IBS, adult patients with IBD demonstrated lower verbal IQ scores, indicating that the disease process of IBD could be related to affect language system. Rolandic operculum was reported to be associated with affective and depressive symptoms for post‐stroke patients (Sutoko et al., 2020). These results could represent an increased cooperation tendency to monitor visceral symptoms and process mental disorders, but it is not easy to establish specific correspondence relationships. The increase in FC between these regions may reflect a positive feedback mechanism for high sensitivity to visceral sensory information, and modulate a neural response to that information, potentially aggravating inflammation.

In our study, there were no significant correlations between the neuroimaging and the duration of the disease. We speculate that the patterns of change in brain activity are related to many factors, not only the duration but also the phenotype of the disease and the frequency of recurrence.

### 4.1 Limitations

The current study has several limitations. First, the sample size was relatively small in our study, despite the estimated sample size in brain imaging being around a dozen subjects (Desmond & Glover, 2002), which can have statistical power analyses. It is necessary to include more subjects in future research for reliable results. Second, it is essential to include more clinical types of diseases and different disease behaviors, and further studies should be conducted to explore the correlation between the clinical manifestations and brain function changes, such as whether the results are consistent in the active and remission stages of diseases, or whether it is reversible between courses. Moreover, the relationship between pain and stress/emotions has not been explored in the current study, and enrollment may have a negative impact on the mood of patients. Finally, multi‐modal fMRI analysis could be conducted in subsequent studies, and longitudinal studies are also required to investigate in subsequent stages.

## 5 CONCLUSIONS

In conclusion, our study showed abnormalities in multiple brain regions of CD patients. Altered ALFF, ReHo, and FC suggested that the involvement of these regions was in the processing of CD through psychological‐neural‐endocrine‐immune regulation. The relationship between specific mechanisms and abnormal manifestations on fMRI needs further study to understand the pathogenesis and provide more information for its clinical diagnosis and treatment.

## AUTHOR CONTRIBUTION

Zong‐Hui Liang designed the experiments, Yan‐Ling Zheng, Qian Xie, Lan Rong collected data, Lu Li and Jie Ma conducted the research, analyzed data and wrote the manuscript. Jian‐Guang Xu supervised the analysis of the data and help language editing. All authors read and approved the final manuscript.

### PEER REVIEW

The peer review history for this article is available at https://publons.com/publon/10.1002/brb3.2243.

## CONFLICTS OF INTEREST

The authors declare no potential conflicts of interest.

## Data Availability

The data that support the findings of this study are available from the corresponding author upon reasonable request.
